# Optimized Extraction of Amikacin from Murine Whole Blood

**DOI:** 10.3390/molecules26030665

**Published:** 2021-01-27

**Authors:** Roccaldo Sardella, Styliani Xiroudaki, Laura Mercolini, Samuele Sabbatini, Claudia Monari, Stefano Perito, Federica Ianni, Anna Vecchiarelli, Stefano Giovagnoli

**Affiliations:** 1 Department of Pharmaceutical Sciences, University of Perugia, Via Fabretti 48, 06123 Perugia, Italy; roccaldo.sardella@unipg.it (R.S.); styliani.xiroudaki@studenti.unipg.it (S.X.); stefano.giovagnoli@unipg.it (S.G.); 2 Center for Perinatal and Reproductive Medicine, University of Perugia, Santa Maria della Misericordia University Hospital, 06132 Perugia, Italy; 3 Department of Pharmacy and Biotechnology (FaBiT), Alma Mater Studiorum, University of Bologna, Via Belmeloro 6, 40126 Bologna, Italy; laura.mercolini@unibo.it; 4 Department of Medicine and Surgery, University of Perugia, Piazzale Gambuli 1, 06132 Perugia, Italy; samuele.sabbatini@gmail.com (S.S.); claudia.monari@unipg.it (C.M.); stefano.perito@unipg.it (S.P.); anna.vecchiarelli@unipg.it (A.V.)

**Keywords:** pharmaco-toxicological investigation, sample derivatization, screening of extraction conditions

## Abstract

Amikacin (Amk) analysis and quantitation, for pharmacokinetics studies and other types of investigations, is conventionally performed after extraction from plasma. No report exists so far regarding drug extraction from whole blood (WB). This can represent an issue since quantification in plasma does not account for drug partitioning to the blood cell compartment, significantly underrating the drug fraction reaching the blood circulation. In the present work, the optimization of an extraction method of Amk from murine WB has been described. The extraction yield was measured by RP-HPLC-UV after derivatization with 1-fluoro-2,4-dinitrobenzene, which produced an appreciably stable derivative with a favorable UV/vis absorption. Several extraction conditions were tested: spiked Amk disulfate solution/acetonitrile/WB ratio; presence of organic acids and/or ammonium hydroxide and/or ammonium acetate in the extraction mixture; re-dissolution of the supernatant in water after a drying process under vacuum; treatment of the supernatant with a solution of inorganic salts. The use of 5% (by volume) of ammonium hydroxide in a hydro-organic solution with acetonitrile, allowed the almost quantitative (95%) extraction of the drug from WB.

## 1. Introduction

Amikacin (Amk) belongs to the class of semisynthetic aminoglycoside antibiotics. It is obtained from kanamycin A by acylation of the C-1 amino group of the 2-deoxystreptamine moiety with L-(-)-γ-amino-a-hydroxybutyric acid [1]. Being refractory to a wide range of aminoglycoside modifying enzymes, Amk is used to treat otherwise aminoglycoside resistant bacterial infections mainly caused by aerobic Gram-negative pathogens such as *Pseudomonas aeruginosa*, *Acinetobacter* spp., and *Enterobacter* spp., as well as mycobacteria and Nocardia [1,2]. Like all aminoglycoside antibiotics, it exerts its antibacterial effect by binding irreversibly to 30S ribosomal subunit and inhibiting thus protein synthesis in a concentration-dependent manner [3]. 

Due to its slight absorption from the gastrointestinal tract, Amk is mainly administered parenterally, either intravenously or intramuscularly [4]. Arikayce, an Amk liposomal suspension for nebulization has been recently approved by the FDA for the treatment of *Mycobacterium avium* complex (MAC) lung disease as a part of a combinational regimen for adult patients who have limited or no alternative options [5]. Amk use is not free from adverse effects, mainly nephrotoxicity and ototoxicity, and consequently optimizing the dose regimen is of crucial importance [1]. 

Being an old drug, anomalous elimination and tissue accumulation of Amk in humans, which could be related to drug partitioning, has already been described [6]. Drug partitioning is often related to sequestration phenomena in tissue and blood cells that can give rise to toxicity concerns due to drug accumulation and secondary interactions [7,8]. Surprisingly, to the best of our knowledge, quantitative analysis of Amk in blood is routinely performed only after extraction from plasma [6,9,10,11,12,13,14,15,16,17,18,19] and no report exists so far on drug extraction from whole blood (WB). This represents an issue as quantification of the drug in plasma does not account for drug partitioning to the blood cell compartment [7], significantly underrating the drug fraction reaching blood circulation. 

In a very recent work [20], we have evaluated the Amk action against hard-to-treat lung infections in a mouse model. For the first time Amk was quantified in murine WB revealing, inter alia, a pronounced binding of the drug to blood components. The observed blood cell binding was never reported before for Amk, despite its high potential clinical relevance. In the present paper, the optimization of the extraction method for Amk disulfate (AmkDS) from murine WB is described. 

## 2. Results and Discussion

A method previously applied by other authors for the extraction of Amk in plasma [21,22] based on the use of sole ACN added to the matrix was tested as the starting point to optimize the process from frozen and sonicated WB samples (Table 1, entry 1). In the present work, the freezing process of the WB samples was functional to a durable storage of the withdrawn WB from mice, while sonication following the thawing of the sample before the extraction, allowed the complete cellular lysis, thereby favoring the release of the drug fraction potentially internalized by the cells. The extraction step was optimized by spiking test WB samples with AmkDS prior to freezing and sonication. 

A well-established HPLC-PDA method already developed by other authors [19] was employed to measure the extraction yield of the scrutinized procedures. Since Amk is a compound with a very weak absorbance within the UV/Vis range, in analogy to [19] the chromatographic method was here implemented with the Amk 2,4-dinitrophenyl derivative. By this way, a favorable UV/Vis absorption was obtained thereby favoring the correct quantitation of the aminoglycoside (Figure S1, Supporting Materials). Pre-column derivatization is a common practice in the HPLC-PDA analysis of compounds lacking suitable chromophore moieties [23,24]. Peak identity in the real sample was confirmed by co-injection with the standard. 

Since the chromatographic part was only at the side of the main purpose of the investigation, we deemed therefore sufficient (in a fit-for-purpose perspective) to rely upon a method previously developed and validated by other researchers [19]. In that case, the method was applied for the accurate dosage of the dinitrophenyl-Amk derivative in biological samples, including plasma, and was validated in terms of linearity, accuracy, precision, lower limit of quantitation, selectivity, recovery, and stability. The very appreciable results reported by the authors underlined the high quality of the validation protocol, thus recommending its use as a reference method.

Several extraction procedures were performed to maximize Amk recovery from WB. The extraction variables were selected based on previous works performed on plasma samples by other authors [9,10,11,12,13,14,15,16,17,18,19], and were iteratively screened according to the heuristic ‘trial and error’ approach. A 5.0 mM aqueous AmkDS solution was always added to a mixture made up of ACN and WB. Ternary mixtures carrying different volumes of the three components were first vortexed to favor the mixing process, and then submitted to centrifugation. None of the obtained supernatants was found to contain Amk (Table 1, entries 1–4). The extraction yield was determined by comparison with the HPLC area value produced by the analysis of a pure AmkDS solution prepared at the same concentration in mobile phase components. 

A strong ‘entrapment’ of AmkDS by the blood cell compartment was therefore readily evident, thus stimulating us to explore further extraction conditions, in which the following parameters were examined: (i) AmkDS solution/ACN/WB ratio; (ii) presence of organic acids for possible membrane protein denaturation and enhanced release of the analyte from blood cell compartments; (iii) presence of NH_4_OH to shift the balance of amikacin towards its undissociated form and potentially favor its extraction; (iv) presence of a buffer system by NH_4_OAc for the possibility of promoting ion pairing and improve extraction yield, while keeping the solvents amenable to MS detection (Table 1, entries 5–17). 

In parallel to the evaluation of different extraction mixtures, also other experimental variables were iteratively screened by following the heuristic approach, focusing the attention on (i) the re-dissolution of the supernatant in water after a drying process under vacuum and (ii) the treatment of the supernatant with a solution of inorganic salts, in order to increase water solubility and dissolution rates by salt, before re-dissolution in water. The use of ionizable additives, including salts and buffers is a common practice to maximize extraction from complex matrices [25]. Very interestingly, the addition of a diluted NH_4_OH solution to the extraction mixture combined to the re-dissolution in water of the dried supernatant obtained after centrifugation, allowed us to partially extract (20.8%) AmkDS from the WB containing mixture (Table 1, entry 16). This result clearly suggested the key role of the basic additive to improve the extraction process, by favoring the balance of basic drugs towards their undissociated form and increase the extraction yield in low polarity solvents. The favorable effect of diluted NH_4_OH solutions for the extraction of basic drugs from WB has been already highlighted by other authors [26]. Only few further attempts were then made to increase the extraction yield, which was maximized (96.2%) by using a relatively more concentrated NH_4_OH solution (5%, *v*/*v*) in the extraction mixture (Table 1, entry 17). Under the identified optimal conditions, the process was repeated twice more, confirming the almost quantitative extraction of Amk from WB (95.5% ± 0.9%). The same extraction method was also applied to four-fold less concentrated test AmkDS solutions, and a highly similar recovery turned out (94.7% ± 0.8%). The extraction yield was markedly lower (11.5%) when the optimized conditions were applied to lyophilized sonicated and frozen WB samples, potentially due to the smaller contact surface between sample and extractant and to the possibility of cell lumps.

The optimized extraction method was also applied to plasma samples [20]. As a result of a strong cell binding, Amk concentration in plasma was found to be extremely low, that is 8.5% [20] of the overall concentration determined in WB. 

## 3. Materials and Methods

### 3.1. Chemicals

Amikacin disulfate (AmkDS) was purchased from Alfa Aesar; HPLC-grade acetonitrile (ACN), ethanol (EtOH) and methanol (MeOH), tris(hydroxymethyl)aminoethane (TRIS), dimethyl sulfoxide (DMSO), 1-fluoro-2,4-dinitrobenzene (F-DNB), ethylenediaminetetraacetic acid (EDTA), ammonium acetate (NH_4_AcO), sodium sulphate (Na_2_SO_4_), sodium chloride (NaCl), acetic acid (AcOH), ammonium hydroxide (NH_4_OH), and trichloroacetic acid (TCA) were purchased from Merck Life Science (Merck KGaA, Darmstadt, Germany) Water for HPLC analysis was purified with a Milli-Q Plus 185 system from Millipore (Milford, MA, USA).

### 3.2. HPLC-PDA Analysis

The HPLC analysis was performed by applying a method previously developed by other authors [19] with only minor modifications. Details are reported as Supplementary Materials.

### 3.3. Whole Blood Samples

WB was withdrawn from female healthy CD1 mice aged 6–7 weeks and weighing 23 ± 2 g (Charles River Laboratories, Wilmington, MA). The animals were housed with a 12-h light/12-h dark cycle. Room temperature was kept constant at 21.1 ± 1.2 °C, while the relative humidity in the interval 40–60%. 

This study was approved by the Academic Ethical Committee and the Italian Ministry of Health (authorization number: 432/2018-PR).

### 3.4. Extraction of Amk from Whole Blood 

The extraction of Amk (as disulfate—AmkDS) was performed on WB samples. Serial blood samples were collected after withdrawal and immediately stored at −80 °C. Then, each sample was subjected to sonication into an ice bath for 5 min. In the optimized extraction process, with the aim to precipitate proteins, an aliquot of 120 µL of blood was mixed with 150 µL of 5% (v) NH_4_OH solution in ACN. The mixture was then vortexed for 30 s and centrifuged at 15000 rpm for 25 min, at a temperature of 5 °C. 

### 3.5. Derivatization of Amk 

The derivatization of Amk was performed according to the method described in [19] with only a few modifications. Details are reported as Supplementary Materials.

## 4. Conclusions

The surprising lack of reliable methods in the literature for Amk quantitation in WB has inspired this work. In fact, Amk determination in plasma cannot effectively highlight drug secondary interactions and partition in the body circulation with potential important clinical implications. 

With the present study, we have demonstrated that the use of 5% (by volume) of ammonium hydroxide in a hydro-organic solution with acetonitrile, is highly effective for the almost quantitative (95%) extraction of Amk from WB. Therefore, our method, by granting good performance and reliability, can represent an additional tool that may enable a more insightful understanding of Amk fate and behavior in the body. Additional studies will be aimed at determining whether the drug blood partitioning observed in mice is also confirmed in humans.

After proper tuning, the proposed method could be expanded to other aminoglycoside antibiotics, likely sharing similar partitioning issues, in turn making possible the evaluation of the possible clinically relevant blood cell binding phenomena.

## Figures and Tables

**Table 1:** Extraction conditions applied in the present study with the corresponding extraction yield.

Entry	Extraction Mixtures ^1^	Other Extraction Conditions	Yield ^2^
1	500 µL AmkDS + 300 µL ACN + 200 µL WB	15000 rpm, 25 min, 5 °C	ND ^3^
2	200 µL AmkDS + 600 µL ACN + 200 µL WB	15000 rpm, 25 min, 5 °C	ND ^3^
3	step 1: 1500 µL AmkDS + 500 µL WB step 2: 200 µL surnatant + 300 µL ACN	15000 rpm, 25 min, 5 °C	ND ^3^
4	10 µL AmkDS + 180 µL ACN + 90 µL WB	15000 rpm, 25 min, 5 °C	ND ^3^
5	40 µL AmkDS + 300 µL ACN + 60 µL WB	step 1: 15000 rpm, 25 min, 5 °C step 2: 200 µL of surnatant dried under vacuum, re-dissolution with 200 µL water	ND ^3^
6	40 µL AmkDS + 300 µL ACN + 0.1% (v) TFA + 60 µL WB	15000 rpm, 25 min, 5 °C	ND ^3^
7	20 µL AmkDS + 600 µL ACN + 20 µL WB	step 1: 15000 rpm, 25 min, 5 °C step 2: 200 µL of surnatant dried under vacuum, re-dissolution with 200 µL water	ND ^3^
8	10 µL AmkDS + 300 µL ACN + 1.0% (v) FA ^4^ + 90 µL WB	step 1: 15000 rpm, 25 min, 5 °C step 2: 100 µL of surnatant dried under vacuum, re-dissolution with 100 µL water	ND ^3^
9	10 µL AmkDS + 300 µL ACN + 1.0% FA (v) ^4^ + 1.0 mM EDTA ^4^ + 90 µL WB	step 1: 15000 rpm, 25 min, 5 °C step 2: 100 µL of surnatant dried under vacuum, re-dissolution with 100 µL water	ND ^3^
10	10 µL AmkDS + 300 µL ACN + 1.0 mM NH_4_OAc ^4^ + 1.0% (v) FA ^4^ + 90 µL WB	step 1: 15000 rpm, 25 min, 5 °C step 2: 100 µL of surnatant dried under vacuum, re-dissolution with 100 µL water	ND ^3^
11	10 µL AmkDS + 300 µL ACN + 10% (v) FA ^4^ + 90 µL WB	step 1: 15000 rpm, 25 min, 5 °C step 2: 100 µL of surnatant dried under vacuum, re-dissolution with 100 µL water	ND^3^
12	10 µL AmkDS + 300 µL ACN + 1.0 mM NH_4_OAc ^4^ + 1.0% (v) FA ^4^ + 90 µL WB	step 1: 15000 rpm, 25 min, 5 °C step 2: 100 µL of surnatant treated with 100 µL Na_2_SO_4_/NaCl (1:4) saturated solutionstep 3: 3000 rpm, 15 min, 20 °C step 4:100 µL of surnatant dried under vacuum, re-dissolution with 100 µL water	ND^3^
13	10 µL AmkDS + 250 µL ACN + 1.0% FA (v) ^4^ + 90 µL WB	15000 rpm, 25 min, 5 °C	12.8
14	10 µL AmkDS + 300 µL ACN + 1.0% (v) TCA ^4^ + 90 µL WB	15000 rpm, 15 min, 5 °C	14.6
15	10 µL AmkDS + 125 µL ACN + 1.0% (v) NH_4_OH ^4^ + 90 µL WB	15000 rpm, 25 min, 5 °C	17.4
16	10 µL AmkDS + 300 µL ACN + 1.0% (v) NH_4_OH ^4^ + 90 µL WB	step 1: 15000 rpm, 25 min, 5°C step 2: 100 µL of surnatant dried under vacuum, re-dissolution with 100 µL water	20.8
17	10 µL AmkDS + 125 µL ACN + NH_4_OH 5% (v) ^4^ + 90 µL WB	15000 rpm, 25 min, 5 °C	96.2

^1^ 5.0 mM Aqueous solution of amikacin disulfate (AmkDS) standard successively added to whole blood (WB); ^2^ Peak Area value %: determined by comparison with the value produced by the analysis of a pure AmkDS solution prepared at the same concentration in mobile phase components; ^3^ Not Detected; ^4^ With respect to the aqueous AmkDS/ACN binary mixture.

## Data Availability

The data presented in this study are available on request from the corresponding author.

## Supplementary Materials

The following are available online: details about the procedure for Amk derivatization; details about the applied HPLC-PDA method. Figure S1: Chromatogram of (A) AmkDS standard solution; (B) an exemplary WB sample; (C) an exemplary WB sample spiked with AmkDS standard.

## Sample Availability

Not available.
