# Supplementary material for: Optimized Extraction of Amikacin from Murine Whole Blood
Source: Molecules. 2021 Jan 27;26(3):665. doi: 10.3390/molecules26030665 (PMC7865403; doi:10.3390/molecules26030665)
Supplement: Supplementary file 1 [file molecules-26-00665-s001.pdf]

Supplementary Materials

Optimized extraction of Amikacin from murine whole blood

Roccaldo Sardella[1,2], Styliani Xiroudaki[1], Laura Mercolini[3], Samuele Sabbatini[4], Claudia Monari[4], Stefano Perito[4], Federica Ianni[1*], Anna Vecchiarelli[4], Stefano Giovagnoli[1]

[1]Department of Pharmaceutical Sciences, University of Perugia, Via Fabretti 48, 06123 Perugia (Italy)

[2]Center for Perinatal and Reproductive Medicine, University of Perugia, Santa Maria della Misericordia University Hospital, 06132 Perugia (Italy)

[3]Department of Pharmacy and Biotechnology (FaBiT), Alma Mater Studiorum, University of Bologna, Via Belmeloro 6, 40126 Bologna (Italy)

[4]Department of Medicine, Medical Microbiology Section, University of Perugia, Piazzale Gambuli 1, 06132 Perugia (Italy)

*Corresponding author:

Dr. Federica Ianni, Department of Pharmaceutical Sciences, Via Fabretti 48, 06123 Perugia (Italy)

federica.ianni@unipg.it

HPLC-PDA analysis

The HPLC study was performed on a Waters ALLIANCE 2695 Separations Module system equipped with a quaternary, low-pressure mixing pump and in-line vacuum degassing, an autosampler with maximum capacity of 120 vials and a column heather/cooler. The system is endowed with a photodiode array (PDA) detector (Waters 2996). The data management was made by a Waters® Millennium®32 Software.

The derivatized samples were analysed according to the following HPLC-UV/Vis method: column, Robusta C18 (250 x 4.6 mm I.D., 5 μm, 110 Å, from SepaChrom, Rho, Italy); mobile phase, ACN/water/AcOH (47:53:0.1, v/v/v); eluent flow rate, 1.0 mL/min; column temperature, 45 °C; sample rack temperature, 20 °C; wavelength of detection, 365 nm; injection volume, 20 μL. After each analysis, the column was always washed with methanol for 15 min at a 1.0 mL/min flow rate. **The selection of the above detection wavelength was done according to the paper by Nicoli and Santi [Journal of Pharmaceutical and Biomedical Analysis 41 (2006) 994–997].**

The physicochemical features of Amk makes this molecule completely soluble in the ternary mixture employed as mobile phase. Moreover, no carry over effect was observed for Amk between consecutive injections. A different situation was instead found for other species whose wash out was complete only with methanol, while only partial with acetonitrile. Therefore, methanol cannot be regarded as a mobile phase component, since it was exclusively used to ensure the complete cleaning of the column, which was always conditioned with the ACN/water/AcOH (47:53:0.1, v/v/v) containing mobile phase before each analysis run.

Before each analysis, the column was equilibrated for 30 min with the selected mobile phase.

A Combitherm-2 CH-3-150 heating/cooling thermostat (from BioSan, Riga, Latvia) was used for the derivatization processes.

Derivatization of Amk

An aliquot of 100 μL supernatant (containing the extracted Amk), obtained by centrifugation, was transferred into a screw-capped tube and dried under vacuum (40 °C). Then, the dried residue was resuspended in 100 μL of water and mixed with 100 μL of 1% (w/v) water solution of TRIS [tris(hydroxymethyl)aminoethane], 200 μL of DMSO (dimethyl sulfoxide) and 200 μL of F-DNB (1-fluoro-2,4-dinitrobenzene) in 95% ethanol (w/v). The tube containing the reaction mixture was vortexed for 30 s and finally incubated at 55 °C in a Combitherm dry-block heater for 40 min. Once cooled at room temperature, the solution was transferred into a micro vial and submitted to HPLC analysis. The standard solutions of Amk (as disulfate – AmkDS) were submitted to the same derivatization procedure.

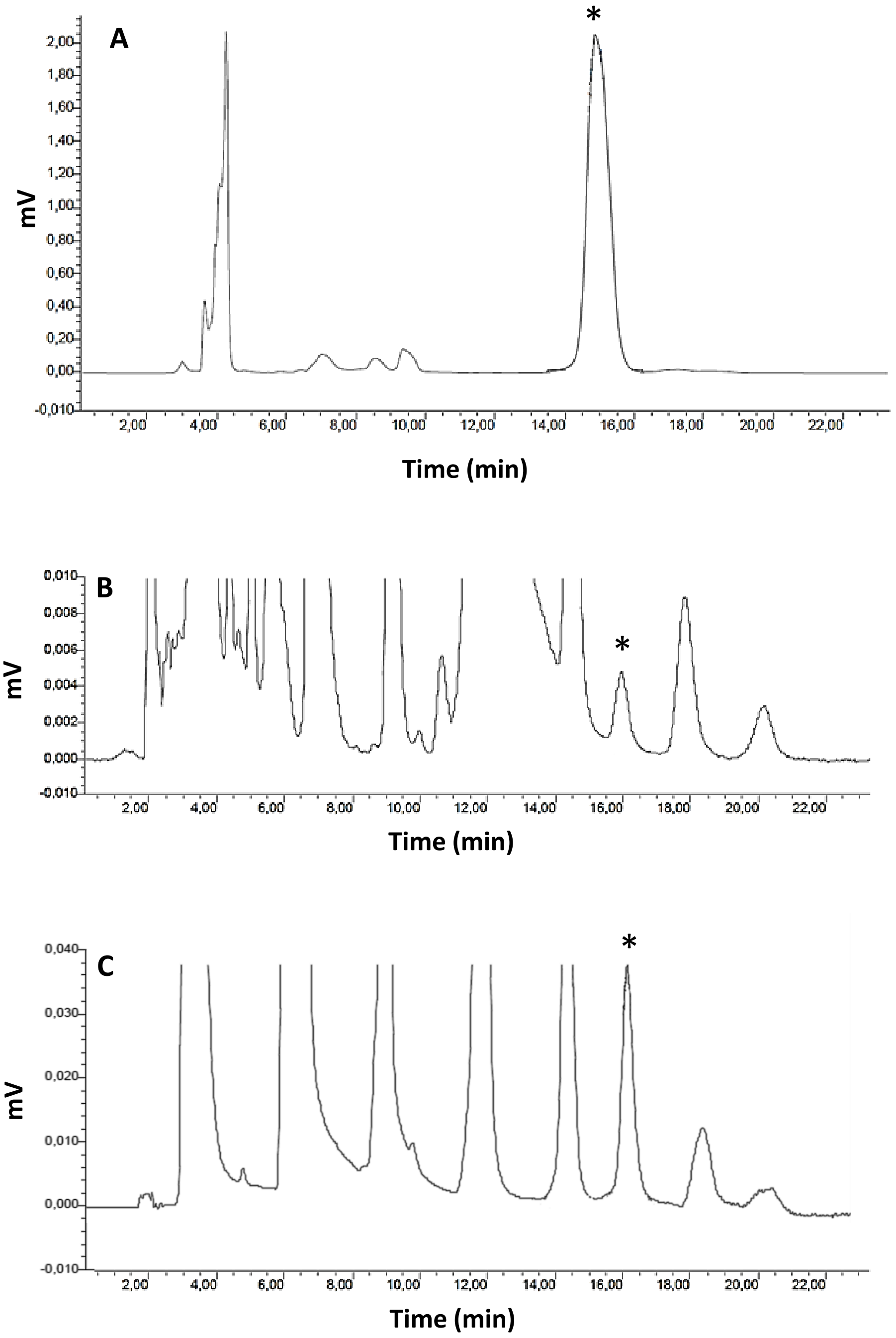

Figure S1. Chromatogram of (A) AmkDS standard solution; (B) an exemplary WB sample; (C) an exemplary WB sample spiked with AmkDS standard. In all cases, the analysis was performed after pre-column derivatization with F-DNB.
